# Decadal Change in Sediment Community Oxygen Consumption in the Abyssal Northeast Pacific

**DOI:** 10.1007/s10498-016-9293-3

**Published:** 2016-05-13

**Authors:** K. L. Smith, C. L. Huffard, A. D. Sherman, H. A. Ruhl

**Affiliations:** 1 Monterey Bay Aquarium Research Institute, Moss Landing, CA USA; 2 National Oceanography Centre, Southampton, UK

**Keywords:** Carbon cycle, Abyssal community, Sediment community oxygen consumption, Long time series, Sediment carbon demand

## Abstract

Long time-series studies are critical to assessing impacts of climate change on the marine carbon cycle. A 27-year time-series study in the abyssal northeast Pacific (Sta. M, 4000 m depth) has provided the first concurrent measurements of sinking particulate organic carbon supply (POC flux) and remineralization by the benthic community. Sediment community oxygen consumption (SCOC), an estimate of organic carbon remineralization, was measured in situ over daily to interannual periods with four different instruments. Daily averages of SCOC ranged from a low of 5.0 mg C m^−2^ day^−1^ in February 1991 to a high of 31.0 mg C m^−2^ day^−1^ in June 2012. POC flux estimated from sediment trap collections at 600 and 50 m above bottom ranged from 0.3 mg C m^−2^ day^−1^ in October 2013 to 32.0 mg C m^−2^ day^−1^ in June 2011. Monthly averages of SCOC and POC flux correlated significantly with no time lag. Over the long time series, yearly average POC flux accounted for 63 % of the estimated carbon demand of the benthic community. Long time-series studies of sediment community processes, particularly SCOC, have shown similar fluctuations with the flux of POC reaching the abyssal seafloor. SCOC quickly responds to changes in food supply and tracks POC flux. Yet, SCOC consistently exceeds POC flux as measured by sediment traps alone. The shortfall of ~37 % could be explained by sediment trap sampling artifacts over decadal scales including undersampling of large sinking particles. High-resolution measurements of SCOC are critical to developing a realistic carbon cycle model for the open ocean. Such input is essential to evaluate the impact of climate change on the oceanic carbon cycle, and the long-term influences on the sedimentation record.

**Electronic supplementary material:**

The online version of this article (doi:10.1007/s10498-016-9293-3) contains supplementary material, which is available to authorized users.

## Introduction

Benthic communities in the deep sea were once thought to be depauperate in life, without discernable external stimuli or temporal variability. However, measurements over the past several decades have shown deep-sea benthic communities in soft-sediment environments exhibit variation at timescales ranging from daily and weekly (Smith et al. 2014) to interannual (Smith et al. 2013). Surface ocean processes, which control the supply of macro- and micronutrients, have long been recognized to cause seasonal and interannual variation in planktonic communities, which are the principal food supply to communities at greater depths and on the seafloor. Not surprisingly, changes in this planktonic food supply are strongly correlated with the structure and activity of deep-sea communities, albeit with a temporal lag on the order of weeks to months (e.g., Smith et al. 2008).

A principal process on the seafloor is the consumption of organic carbon (food demand) by the benthic community, which is fueled by sinking particulate organic carbon (POC, food supply) entering the benthic boundary layer from overlying waters (e.g., Jahnke 1996; Glud 2008). A critical research focus has been the balance, or lack thereof, between food supply and demand by the benthic community (Smith and Kaufmann 1999). In time-series studies, measurements of sediment community oxygen consumption (SCOC) are used as a proxy for organic carbon consumption, while sinking POC flux has been used to estimate food supply.

Some of the early estimates of the balance of food supply to demand suggested that there was an unexpected deficit in food supply in the northeast Pacific (Smith 1987), which served as a motivation in developing more detailed time-series estimates. As time-series records developed, there were times when food both supply and demand seemed to balance (Smith et al. 1992) and when substantial deficits of food supply persisted (Smith and Kaufmann 1999). Similarly, there were other relatively oligotrophic regions where SCOC dynamics appeared somewhat stable (Sayles et al. 1994) or showed clear seasonal variation (Smith et al. 2002). Overall, these results left a very puzzling view of organic carbon remineralization over much of the world’s seafloor.

Our objective here is to present a 27-year time series of SCOC measurements using a variety of autonomous and observer-directed instruments to show how carbon supply and demand at an abyssal soft-sediment community have changed over time. A long time-series study of an abyssal community was initiated in 1989 at Sta. M, off the central California coast, to monitor changes in seafloor processes on decadal timescales (Smith and Druffel 1998). Despite the significance placed on deep-sea carbon sequestration and ocean acidification in policy discussions (e.g., Levin and Le Bris 2015), to our knowledge, our dataset represents the only long time-series effort to directly quantify carbon supply and remineralization processes on the abyssal seafloor.

Two of the primary measurements of this time series were SCOC and POC flux. POC flux has been measured throughout this time series using sequencing sediment traps, typically with 10-day resolution. We present data from four different instruments used to measure sediment community oxygen consumption in situ over a 27-year time series. There was a long hiatus in SCOC quantification between 1999 and 2005 due to a lapse in funding. A shorter hiatus occurred in 2008–2009 due to unavailability of ship-time. A free vehicle grab respirometer (FVGR) was deployed during seasonal cruises to Sta. M between June 1989 and June 2012. Tube core respirometers (TCRs) were deployed in August and September 1994 and April and May 1995. Two different designs of benthic rovers have also been extensively used during the time series. Rover I was deployed continuously between February and May 1996, and Rover II has been deployed nearly continuously starting in May 2011 and remains in service. The short-term measurements using the FVGR and TCR provided useful daily scale snapshots of sediment community activity, but lacked the temporal resolution of the POC flux measurements. This sampling asymmetry meant that significant changes in the food supply could occur during periods when no SCOC measurements were available, precluding high-resolution investigation of food supply and demand budgets. To alleviate this problem, we then developed a bottom transecting autonomous vehicle (Rover I) that could transit the seafloor between servicing cruises and measure SCOC on a comparable temporal scale as the food supply measurements (Smith et al. 1997). Rover I was lost during a mission in the central Pacific in 1999. Rover II development began shortly afterward, and this vehicle has been in operation since 2011 (McGill et al. 2009; Sherman and Smith 2009; Smith et al. 2013, 2014). Synoptic measurements of SCOC between many of these instruments have yielded comparable fluxes with no significant differences (Smith et al. 1997, 1998, 2013; Smith and Kaufmann 1999), allowing for long-term changes to be assessed.

Over the past 27 years, changes in SCOC at Sta. M have correlated with changes in particulate organic carbon supply from the overlying water column, ultimately connecting the surface ocean conditions to the abyssal seafloor (Smith et al. 2009). For much of the record, however, it seemed there was insufficient food supply, even accounting for visible depositions of particulate organic matter on the seafloor (e.g., Smith et al. 2008). However, observations in 2011 and 2012 of new peaks in POC flux and subsequent SCOC suggested that large-scale food depositions that occur as rarely as once a decade may aid in balancing demand (e.g., Smith et al. 2014). Here, we use newly acquired data extending into 2015 to explore the daily, seasonal and long-term variation in POC flux and SCOC, and discuss the potential for balancing these rates in terms of carbon cycle dynamics.

## Area of Investigation

**Fig. 1:**
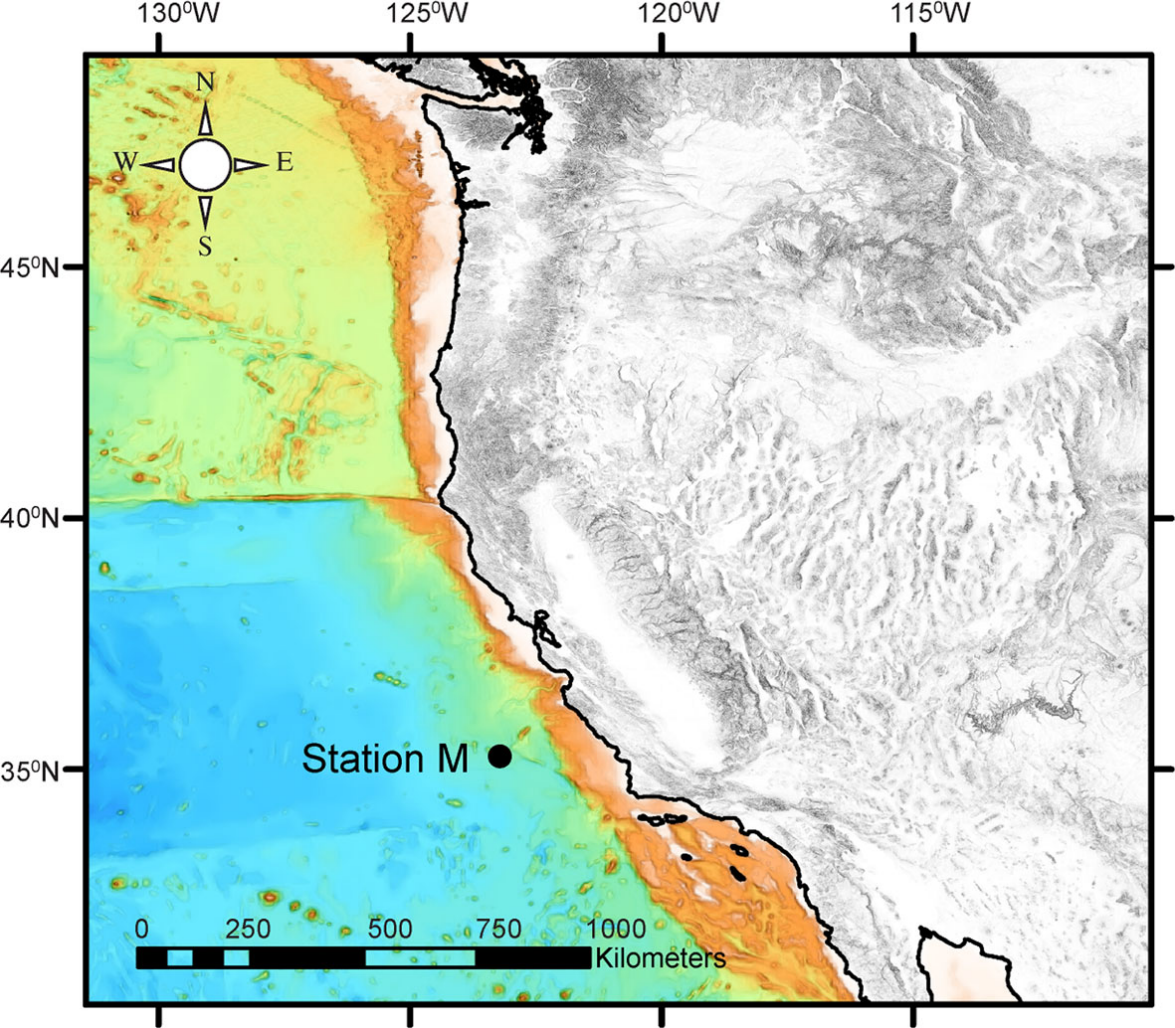
Location of Station M in the northeastern Pacific. Figure prepared with ArcMap 10.1, Bathymetric layer: http://services.arcgisonline.com/ArcGIS/servicesOcean_Basemap2013

Station M is an abyssal long time-series site located approximately 200 km west of the central California coast on the Monterey Deep-Sea Fan at a depth of 4000–4100 m (Fig. 1; Smith and Druffel 1998). This soft-bottom area is characterized by silty clay sediments with low topographic relief (<100 m change in elevation over a 20 km^2^ area). This station was chosen to represent an abyssal area underlying the California Current with strong seasonal upwelling and productive headland plumes frequently projecting offshore over the site. The surface chlorophyll between 1998 and 2013 has ranged from 0.2 to 1.2 mg m^−3^, while the estimated net primary production over the same period went from a winter low of 282 up to a spring maximum of 1001 mg C m^−2^ day^−1^ (Smith et al. 2013, 2014). The seafloor is occupied by mobile megafauna such as holothurians and echinoids which serve as bioturbators of the sediment (Vardaro et al. 2009), and conspicuous stationary fauna such as sponges and polychaete tubes projecting into the bottom water (Beaulieu and Smith 1998; Kuhnz et al. 2014; Lauerman et al. 1996). The sediment is punctuated with burrow openings and mounds of biological origin. Bottom currents have a semidiurnal tidal influence with principal components being northwest and southeast. The mean current speed is 2 cm s^−1^ with a range from 0 to 25 cm s^−1^ (Beaulieu and Smith 1998). Dissolved oxygen concentration in the bottom water remains fairly constant at 135.6 ± 2.6 μmol L^−1^. Turbidity in the bottom water reaches peaks during seasonal sedimentation events of planktonic origin primarily in the late spring into the fall months. The sinking flux of particulate organic carbon ranges two orders of magnitude from 0.3 to 32 mg C m^−2^ day^−1^ with the highest inputs between late spring and mid-fall. A large portion of this material was composed of upper water column plankton exuviae, fecal pellets and carcasses (Wilson et al. 2013). The sediment community infauna is largely composed of foraminifera, nematodes, nemerteans, polychaetes and crustaceans (Drazen et al. 1998; Laguionie-Marchais et al. 2013; Ruhl et al. 2008). The microbial community is dominated by Crenarchaeota (Moeseneder et al. 2012) and Gammaproteobacteria (Kouridaki et al. 2010).

## Methods

### SCOC Measurements

#### Free Vehicle Grab Respirometer

**Fig. 2:**
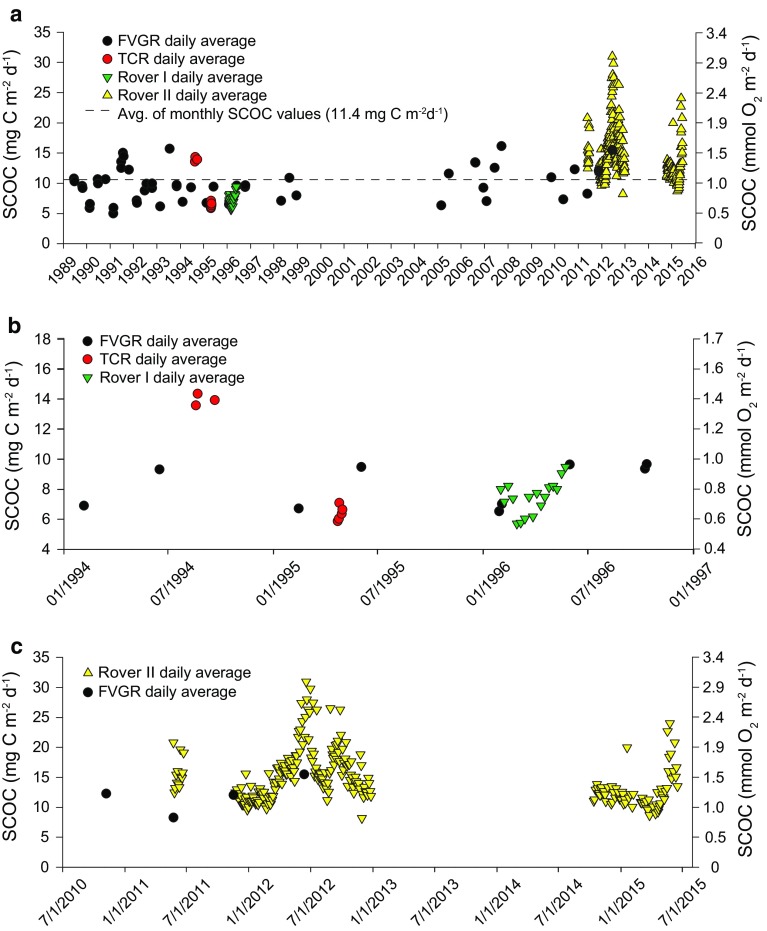
Daily averages for all instruments measuring SCOC at Sta. M [*black dots* free vehicle grab respirometer (FVGR), *red dots* tube core respirometer (TCR), *green triangles* Rover I, *yellow triangles* Rover II, *black dashed line* average of all daily values] **a** spanning the full time series (June 1989–June 2015), **b** January 1994–January 1997 to show overlapping measures by FVGR, TCR and Rover I, and **c** July 2010–July 2015 showing overlapping measures by FVGR and Rover II

The first instrument to measure SCOC at Station M was the free vehicle grab respirometer (FVGR), an autonomous tripod or tetrapod frame with attached flotation, four stainless steel respiration chambers, tandem acoustic releases and disposable steel ballast (Smith and Baldwin 1983; Smith et al. 1979; Smith 1987). FVGR was deployed for 2- to 3-day incubations to yield short-term measurements of SCOC, made on a roughly seasonal basis beginning in June 1989 and ending in June 2011, with a total of 51 deployment periods (Fig. 2a). This instrument measured oxygen consumed by enclosed sediment and overlying water in four grab chambers (Smith 1987). It was modified over the course of 20 years in service to minimize disturbance on seafloor landing and increase reliability of the oxygen measurements by switching from polarographic to optode sensors. The four respirometer chambers were mounted centrally on a rigid open framework (tray) held above the sediment surface to reduce initial disturbance caused by the tripod landing on the seafloor. Each chamber enclosed a seafloor area of 413 cm^2^ and up to 15 cm of overlying water (Smith et al. 2001). Following landing on the seafloor, a pair of hydrostatic pistons mounted on either side of the respirometer tray was timer actuated to slowly push the four grabs synchronously into the sediment to a depth of ~15 cm. The water volume in each grab was estimated from the measured height of the overlying water minus the displacement volume of the stirring mechanism. This insertion depth left ~15 cm of overlying water in each chamber (Smith and Baldwin 1983). Later versions of the FVGR replaced the hydrostatic pistons with a single motor-driven piston, which was mounted centrally on the tray to avoid synchronization issues associated with using the two-piston design. A stirrer was inserted in the top of each chamber to maintain homogeneous mixing of the overlying water while the oxygen concentration was monitored using polarographic sensors, which were later replaced with more reliable Aanderaa optodes. An electronic timer was programmed to actuate all the functions including respirometer placement, water sampling and the final grab closure before acoustically signaling the release of the ballast weight. The positively buoyant FVGR then ascended to the surface for shipboard recovery.

#### Tube Core Respirometers

The second approach to measuring SCOC was to use the manipulative capabilities of the DSRV Alvin and remote controlled vehicles (ROVs) to place tube core respirometers (TCRs) over visually selected areas of the seafloor. These individual respirometers consisted of an acrylic tube beveled on the open end and capped on the other with a stirring motor, polarographic sensor, electronic controller and alkaline battery pack (Smith and Druffel 1998). Each core enclosed a 38.4 cm^2^ area of the sediment surface to a depth of 20 cm leaving 15 cm of overlying water. The final water volume in each core was estimated from the measured height of the overlying water minus the displacement volume of the stirring mechanism (see Smith and Druffel 1998). Approximately 2-day incubations were made before the TCRs with enclosed sediment were recovered and processed aboard ship. In August and September 1994 and in April 1995, three series of intensive measurements of SCOC were conducted using DSRV Alvin manipulated TCRs (Fig. 2a, b). Some of the TCRs were placed over detrital aggregates, while others were placed in areas devoid of visible aggregates (Smith and Druffel 1998). Here, we assume that both areas resulted in representative SCOC values, and so they are not differentiated in further time-series analyses.

#### Rover I

The first Rover (I) was developed with glass sphere flotation mounted in racks above the tractor-like chassis with acoustically controlled releases and a disposable steel weight slung between the two treads (Smith et al. 1997). On the front of the Rover was an instrument rack mounted on two lead screws for lowering two acrylic benthic chambers into the sediment. Each chamber enclosed ~730 cm^2^ of sediment surface with the oxygen concentration monitored with a polarographic oxygen sensor in a flow cell. A stirring assembly circulated the chamber water to minimize stratification. Video cameras were mounted obliquely on the instrument rack to record the insertion of the chambers and depth of penetration into the sediment. A survey time-lapse camera was mounted on the front of the Rover to provide images of the seafloor during transits between measurement sites. During a 5-month deployment at Sta. M from January to May 1996, Rover I measured SCOC at 17 sites with 152-h incubations along a transect moving either up or across current to avoid disturbance to each measurement location (Fig. 2a, b; Smith et al. 1997).

#### Rover II

A second-generation Benthic Rover (II) was developed, similar in design to the prototype. Rover II is an autonomous track vehicle that transits a pre-programmed transect up or across current along the seafloor making measurements of SCOC for deployments up to one year (Sherman and Smith 2009; Smith et al. 1997). The principal changes incorporated into Rover II included syntactic foam flotation and an independent rack system for each of the two benthic chambers (Sherman and Smith 2009). The oxygen sensors were updated to use Aanderaa optodes (Model 3830) for chamber and reference measurements. Three pump-actuated valves were mounted into the top of each chamber and actuated during insertion at each site to prevent bow-wave disturbance. Rover II has been deployed routinely at Sta. M from April 2011 until present except for a 1.5-year gap of non-function between 2013 and fall 2014. Cameras imaged the insertion depth of each chamber into the sediment and any enclosed sediment features including megafauna and burrows. A total of 250 daily averages of SCOC (from 416 chamber measurements; Fig. 2a, c) have been made with Rover II, affording an opportunity to evaluate high temporal resolution SCOC compared to synoptic POC flux measurements with sediment traps in the overlying water.

### Sinking POC Measurements

**Fig. 3:**
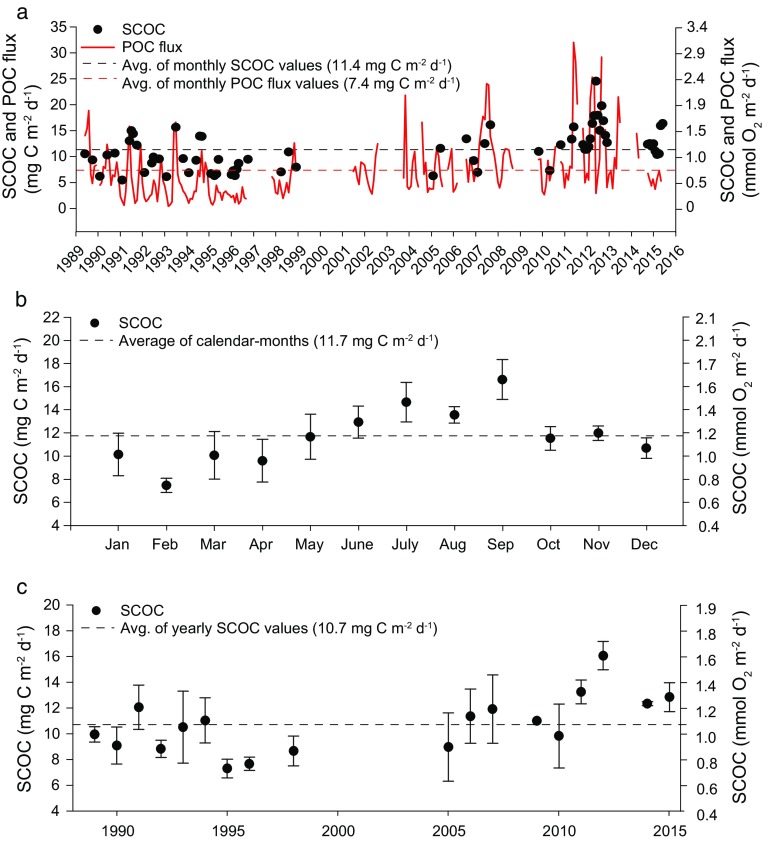
SCOC (*black dots*) **a** monthly averages with POC flux (*red solid line* POC flux, *red dashed line* average of all monthly POC flux values, *black dashed line* average of all monthly SCOC values), **b** averages per month ± SE (*black dashed line* average of calendar-month values), and **c** year ± SE (*black dashed line* average SCOC of yearly averages)

Sinking particulate matter was collected in funnel-shaped sequencing sediment traps with a sampling frequency of 10 days at 600 and 50 m above bottom (Smith et al. 1994). Sediment traps with a 0.25 m^2^ collection area and a 13-sample cup sequencer were deployed from 1989 to 2010 (Baldwin et al. 1998). Subsequently, we used sediment traps with a 0.50 m^2^ collection area and a 21-sample cup sequencer (McLane Research Laboratories, model Particle Flux Mark 78H-21). Sample cups were filled with poison prior to deployment (3.0 mmol L^−1^ HgCl_2_, from 1989 to 2009; 3 % buffered formalin from 2009 to present). Swimmers (organisms that swam rather than sank into the traps) were removed from all samples after recovery. Sediment samples were frozen at −20 °C. In the laboratory, samples were thawed and split with three-quarters of each sample analyzed for organic carbon following the procedure described by Smith et al. (2014). POC flux from the 600-mab sediment trap is presented where available (Fig. 3a). Gaps in the 600-mab POC flux record were infilled, where possible, with values from the 50-mab trap using the linear relationship between these two time-series datasets [POC flux _600mab_ = 3.2 ± (0.44 × POC flux_50mab_)].

### Data Analyses

SCOC data were recorded as a daily average associated with the initial date of each incubation. All oxygen consumption measurements are presented in mmol O_2_ m^−2^ day^−1^. These values were then converted into organic carbon using a respiratory quotient of 0.85 for mixed carbohydrate and lipid with final units given as mg C m^−2^ day^−1^ (Smith et al. 1979). Synchronous deployments of SCOC monitoring instrumentation have confirmed the resulting measurements do not differ significantly, and can be compared in analyses of seasonal and yearly patterns in SCOC [FVGR and TCR compared in Fall 1994 and Spring 1995; FVGR and Rover I compared in Spring 1996; FVGR and Rover II compared in Fall 2011 (Smith and Druffel 1998; Smith et al. 1997, 2013)]. SCOC and POC flux were averaged for one-month periods (referred to as “monthly average”). To examine seasonal patterns, monthly averages were binned by “calendar-month” (e.g., January, February, March). SCOC measurements made with the four different instruments were combined for all averages at the monthly level to avoid overweighting of high-resolution data. In order to examine the potential influence of temporal resolution on our results, we also examined the Rover II data at 10-day scale in terms of correlation between POC flux and SCOC. We also examined a daily scale integrated estimate of POC flux:SCOC. Graphs were prepared in SigmaPlot 10.0. Statistical analyses were performed using StatXact 4.0.1

## Results

### Instrument Comparison

Daily SCOC measurements throughout the 27-year time series using four different instruments ranged from a low of 5.0 mg C m^−2^ day^−1^ in February 1991 to a high of 31.0 mg C m^−2^ day^−1^ in June 2012 (Fig. 2a). Monthly averages of SCOC measured by all instruments are presented in Supplementary Table 1. The full time-series average of all monthly averages was 11.4 mg C m^−2^ day^−1^. TCR-based SCOC, from tubes placed over detrital aggregates, was significantly higher in August and September than rates measured with the randomly deployed FVGR in June 1994. TCR measurements of SCOC made in April 1995 were of comparable magnitude to FVGR rates in February 1995 but lower than rates measured 2 months later in June.

The Rover I deployment from January to May 1996 yielded the first contiguous measurements of SCOC at 17 sites along a continuous transect with duplicate chambers at 13 of the sites. SCOC measured during this deployment showed considerable seasonal variation dropping to a low in March of 5.7 mg C m^−2^ day^−1^ and then consistently rising to a high in May of 9.5 mg C m^−2^ day^−1^ (Fig. 2b). FVGR deployments made at times coinciding with both ends of this Rover time series agreed to within 2 mg C m^−2^ day^−1^ with concurrent Rover measurements.

Rover II was first deployed at Sta. M in Spring 2011 and conducted measurements of SCOC along a transect of 13 sites (Fig. 2c). During the first week of Rover II measurements in late May 2011, SCOC rates were twice as high as the average of four FVGR measurements of SCOC in the previous week. After a 5-month hiatus, Rover II was deployed continuously from November 2011 until December 2012. During this one-year deployment, SCOC ranged from a low of 8.2 mg C m^−2^ day^−1^ in November to a high of 31.0 mg C m^−2^ day^−1^ in June 2012, the highest rate ever measured at Sta. M. SCOC measurements made with the FVGR at the beginning and midway through the Rover deployment were comparable in magnitude (Fig. 2c). Rover II was again deployed in Fall 2014 and conducted a series of 68 SCOC measurements until June 2015 when it was recovered for servicing and redeployment. SCOC remained relatively consistent for the majority of this deployment until early spring when rates rose precipitously to 24.0 mg C m^−2^ day^−1^, returning to higher rates earlier in the seasonal cycle than in previous years.

### Seasonal Variation in SCOC

SCOC exhibited seasonal variation, with highest values recorded during September (16.6 mg C m^−2^ day^−1^) and lowest during February mg C m^−2^ day^−1^ (7.5; Fig. 3b). Standard error of the mean for each calendar month did not show a seasonal pattern, fluctuating between 0.6 mg C m^−2^ day^−1^ and 2.1 mg C m^−2^ day^−1^ throughout the year.

### Interannual Variation

SCOC monthly averages ranged between 5.5 and 16.1 mg C m^−2^ day^−1^ over the first 20 years of the time series (Fig. 3a), and 7.3 and 24.6 mg C m^−2^ day^−1^ over the next 6 years. The full time-series SCOC average of all 12 calendar-months was 11.7 ± 0.7 mg C m^−2^ day^−1^ (±SE, *n* = 12 mo). From 1989 to 2009, the SCOC calendar-month average was 9.6 ± 0.9 mg C m^−2^ day^−1^ (±SE, *n* = 12 mo). From 2010 to 2015, the SCOC calendar-month average was 14.5 ± 0.8 mg C m^−2^ day^−1^ (±SE, *n* = 12 mo). Calendar-month average SCOC was significantly higher in the latter period of the time series (Wilcoxon signed rank *p* = 0.002). With increased temporal resolution provided by Rover II starting in 2011, higher- and shorter-interval peaks were measured. Monthly averages were higher especially in 2012 when mean SCOC peaked at 20.4 mg C m^−2^ day^−1^.

Yearly averages were calculated by averaging calendar-month values (avg. 4 per year). These values were not distributed consistently throughout the seasons. Yearly averages of SCOC exhibited considerable variation throughout the time series with the lowest rate in 1995 of 7.3 mg C m^−2^ day^−1^ (Fig. 3c). The highest annual SCOC was measured in 2012 (16.0 mg C m^−2^ day^−1^).

The full time-series POC flux average of all 12 calendar-months was 7.4 ± 0.6 mg C m^−2^ day^−1^ (±SE, *n* = 12 mo). From 1989 to 2009, the average POC flux of all 12 calendar-months was 6.5 ± 0.6 mg C m^−2^ day^−1^ (±SE, *n* = 12 mo). From 2010 to 2015, average POC flux of all 12 calendar-months was 10.5 ± 1.5 mg C m^−2^ day^−1^ (±SE, *n* = 12 mo). Average POC flux was significantly higher in this latter period (Wilcoxon signed rank text *p* = 0.01). POC fluxes reached an unprecedented rate of 32.0 mg C m^−2^ day^−1^ in June 2011 with another peak of 29 mg C m^−2^ day^−1^ in September 2012, about fourfold the yearly average.

### Comparison of SCOC and POC Flux

**Fig. 4:**
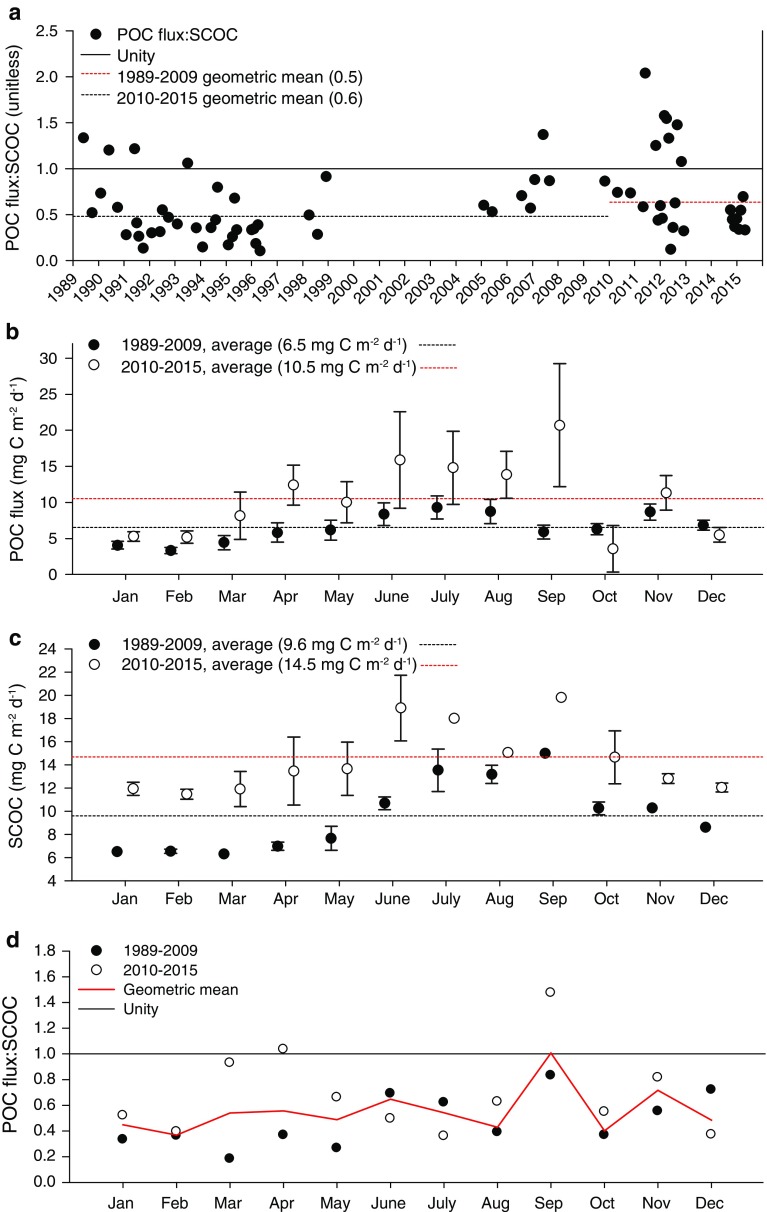
Monthly averages of **a** POC flux divided by SCOC (*black dots* POC flux:SCOC; *solid black line* unity), **b** each calendar-month’s average of POC flux ± SE, **c** SCOC ± SE, and **d** POC flux:SCOC (*solid black line* unity, *solid red line* calendar-month geometric mean). In *panels* **b**–**c** *black dots* 1989–2009 values, *white circles* 2010–2015 values, *solid black line* unity, *black dotted line* 1989–2009 geometric mean or average (indicated in *figure*), *red dotted line* 2010–2015 geometric mean or average (indicated in *figure*)

Monthly means of SCOC generally reflected the peaks and valleys in POC flux. Monthly averages of SCOC and POC flux correlated significantly with no time lag (Spearman *ρ* = 0.67, *p* < 0.0001). The highest values for both datasets were recorded in 2011 and 2012 (Fig. 3a). POC flux:SCOC ranged from 0.11 in May 1996 up to 2.0 in June 2011 (Fig. 4a). Twelve of 66 monthly ratios exceeded unity (Fig. 4a). Both POC flux (Fig. 4b) and SCOC (Fig. 4c) showed seasonal patterns, and monthly averages that were lower from 1989 to 2009 compared to 2010 to 2015. There was no seasonal pattern in monthly POC flux:SCOC ratios, and so each individual monthly value was included in the geometric mean (Fig. 4d). The geometric mean of all of POC flux:SCOC monthly ratios was 0.5 (unitless). POC flux:SCOC ratios were higher from 2010 to 2015 (geometric mean = 0.6, *n* = 25) compared to 1989–2009 (geometric mean = 0.5, *n* = 41), although these measurements were highly variable (Mann–Whitney *U* = 378.0, *p* = 0.04, Fig. 4b, c). This summary is limited to months for which both POC flux and SCOC monthly averages were available. For years with both POC flux and SCOC flux measurements, the yearly average POC flux accounted for 63 % of the estimated carbon demand of the benthic community (yearly average of SCOC).

The Rover II resolved SCOC at 10-day intervals allowing time lagged correlations with POC flux. These correlations peaked at *ρ* = 0.65 with a 61-day time lag between POC flux and SCOC. The 10-day integrated rate of increase for POC flux peaked at 3.55 mg C m^−2^d^−2^ in June 2011, while SCOC reached a maximum rate of increase of 0.55 mg C m^−2^d^−2^ in May 2015. When examining the 10-day POC flux:SCOC for these data, the food supply accounted for 67 % of the demand.

## Discussion

### Long-Term Carbon Balance

Long time-series studies of sediment community processes, particularly sediment community oxygen consumption, have shown similar fluctuations with the flux of POC reaching the abyssal seafloor. SCOC quickly responds to changes in food supply (Smith et al. 2014), and tracks POC flux over the long term (Fig. 3a). Yet, SCOC demands consistently exceed POC flux supply as measured by sediment traps alone. Over a 27-year period, the POC flux has been insufficient to meet the estimated organic carbon demands of the sediment community at Sta. M. However, the discrepancy of 37 % could reasonably be explained by sediment trap sampling artifacts with the important implication that over decadal scales, at least, POC flux:SCOC is probably balanced.

Major deposition events at Sta. M such as a flux of salp tunics and fecal pellets have increased the POC flux over a 6-month period in 2012, to exceed SCOC up to 327 % (Smith et al. 2014). Large pulses of organic carbon such as salps were underestimated by sediment traps and probably form a significant food input to the benthic community to offset any deficiencies over extended periods of time (Smith et al. 2014). Exclusion due to baffle size, clogging and the influence of undersampling by sediment traps, lateral advection of marine and terrestrial organic matter in the benthic boundary layer from the continental margin could each contribute to underestimation of POC flux with sediment traps (e.g., Smith et al. 2001, 2014).

Undersampling of sinking particulate matter by conventional sediment traps has long been recognized as a bias. Concurrent sampling of phytodetritus in a sediment trap moored at 50 m above bottom and collections on the seafloor were different in taxonomic proportions at Sta. M (Beaulieu and Smith 1998) but similar in chemical composition (Smith and Druffel 1998). Peaks of visible POC accumulation on the seafloor not accounted for in sediment traps can be found throughout the time series (Smith et al. 2008, 2014). A salp bloom and subsequent sedimentation event in 2012 covered up to 98 % of the seafloor with salp tunics, estimated from time-lapse camera images. However, few salp tunics were collected concurrently in sediment traps at 50 m above bottom (Smith et al. 2014). Large mucous houses produced by planktonic larvaceans can be up to 1 m across (Robison et al. 2005) and sinking through the water column they can easily clog sediment trap baffles and/or cup openings causing underestimates of particle flux (Baldwin et al. 1998). The contribution of organic carbon input from larvacean houses to the deep-sea benthic community can account for a substantial input of organic carbon (Robison et al. 2005). Radionuclide studies with ex^230^Th showed sediment trap fluxes underestimated the sediment accumulation by an order of magnitude at Sta. M (Shaw et al. 1998). However, this difference can be reconciled by deposition events of large detrital aggregates (Shaw et al. 1998).

Lateral advection of material from the continental margin to Sta. M has been indicated by lower radiocarbon content of the suspended material below 2500 m depth (Druffel et al. 1998). High concentrations of pyrophaeophoride a, a degradation product of Chl a, were found in the benthic boundary layer, suggesting lateral advection (Bianchi et al. 1998). A spike in the C:N of the POC flux up to 84.8 in 1995 at 50 m above bottom at Sta. M indicated an influx of terrestrial material (Smith et al. 2001). Episodic inputs of organic matter to Sta. M from the continental margin cannot be discounted.

Given the biases associated with measurements of SCOC and POC flux at Sta. M, there is reasonable agreement between the organic carbon supply and the carbon demand of the sediment community over the 27-year time series. However, reaching this conclusion has only been possible through a long time-series study of pelagic and benthic processes. The earlier assessments of balance between carbon supply and demand revealed a significant discrepancy in the POC flux which was greatest in the mid-1990s (Smith and Kaufmann 1999). Over this period, the POC flux was estimated to contribute only 52–59 % of the demand by the sediment community. This deficit continued through 2007 (Smith et al. 2009). However, there was a rebound in the POC flux between 2011 and 2013 with unprecedented sedimentation events of detrital aggregates and salp detritus (Smith et al. 2013, 2014). After a hiatus in measurements in 2014, POC flux:SCOC declined sharply below unity again (Fig. 4a). This long-term variation in the supply and demand of organic carbon in the deep ocean at Sta. M appears dependent on episodic fluxes of particulate matter to the seafloor from surface waters. Such episodic pulses may serve to sustain the benthic community over long periods of reduced supply. Direct evidence of these pulses can be short-lived, but the excess supply can be sufficient to sustain the community during long-term deficits.

### Spatial Heterogeneity

The issue of spatial heterogeneity in SCOC measurements has been addressed at Sta. M regarding the presence of detrital aggregates (Smith et al. 1998) and the presence of megafauna (see Sect. 5.3). Small-scale changes in diffusive oxygen uptake on the scale of a few cm were measured in Sagami Bay at a depth of 1450 m. Such small-scale changes would be included in the larger area measurements made with the FVGR or Rover chambers. To evaluate the importance of spatial variation in SCOC, we used the paired port and starboard measurements on Rover II over a year long period, November 21, 2011 through December 26, 2012, while removing those measurements noted with megafauna. There was no significant difference between measurements by the paired chambers using a paired *t* test (*t* = 0.467, df = 157, *p* = 0.64). On a spatial scale of ~730 cm^2^ at Sta. M, there is no indication of a significant influence of spatial heterogeneity on SCOC.

### Megafauna Influence

It has been well recognized that SCOC measurements from chambers systematically discount the food demand of larger fauna that are less densely distributed and therefore less likely to be included in chamber measurements. The Rover system importantly makes many more measurements over time than landers. About 10 % of the Rover measurements were recorded when megafauna (about 1 cm in size or larger) were visible in the chamber. Comparing the variation in chamber measurements containing megafauna to the synoptic chamber estimates without megafauna gives an opportunity to estimate the impact of megafauna on SCOC. During the period November 2011 to December 2012 when both chambers were working well over a full year, only two-thirds of the chambers with megafauna had values that were higher than the synoptic paired estimate and fewer than half had values that exceeded 20 % of the synoptic estimate. While megafauna respiration might occasionally augment an SCOC estimate, their contribution is considered here as part of the overall sediment community carbon demand. We examined the influence of these megafauna by removing all chamber estimates that enclosed megafauna and exceeded 20 % of the synoptic chamber SCOC measurement. This removal made approximately a 2 % difference in the deficit between food supply and demand over that period.

### Integration and Rates of Change

There is considerable scientific benefit to measuring SCOC at high temporal resolution. Peaks in both SCOC and POC flux are short-lived and can be missed partially or fully by seasonal sampling. Likewise, because the timing and magnitude of these high-sedimentation events is not consistent, sampling gaps caused by uneven sampling throughout the months of the year are problematic for calculating long-term change in POC flux:SCOC, and estimating carbon budgets. We cannot currently fill gaps in FVGR values based on models with sufficient confidence to be useful in balancing food supply and demand at a monthly scale. Calculations of undersupply for the periods represented only by FVGR and TCR measurements were highly sensitive to the averaging method used. Full integrations of carbon supply and demand were only available starting in 2011, when Rover II SCOC temporally matched POC flux measurements for an entire yearly cycle. These high-resolution measurements are critical to carbon budget estimates at abyssal depths.

SCOC has been known to respond quickly to inputs of POC both from long-term measurements (e.g., Smith et al. 2014) and short in situ experiments (e.g., Sweetman and Witte 2008). However, the observed acceleration/deceleration in SCOC is about an order of magnitude less than that of POC flux providing insight into the longer-term integration of carbon dynamics in the SCOC record than that of the more variable POC flux. An advantage of the Rover system is its near-continuous record of SCOC to provide finer temporal resolution in attempts to balance the time integrated food supply and demand. Looking at the November 2011 to December 2012 period, which has the longest continuous record of both sediment trap and Rover data, provided an opportunity to look at this balance in more detail. Over that same period, the average balance of food supply to demand was 0.68 at 10-day resolution, the same as for the whole Rover II record. When examining 10-day integrated variation, the correlation between SCOC and POC flux is highest with a time lag of 61 days.

The seafloor is, in effect, a sediment trap. High-resolution temporal measurements of SCOC have clear value in developing a realistic carbon cycle model for the open ocean. Such input is essential to evaluate the impact of climate change on the oceanic carbon cycle, and the long-term influences on burial rates and the sedimentation record. Jahnke (1996) made a global estimation of seafloor oxygen flux using sedimentary organic carbon, CaCO_3_ and accumulation rates. Our study using SCOC as an estimator of carbon dynamics has shown undersampling and high temporal variance in sediment trap derived POC flux relative to SCOC variation. There are potentially important implications using sediment trap data if they indeed under sample POC flux on the order of 37 %. If true, then larger and faster sinking material is likely to be relatively dominant in making up the “missed” POC. So, the vertical transfer rate formula terms used in climate models should be interpreted with undersampling in mind. The use of image-based estimations of carbon dynamics is a particularly promising avenue for augmenting sediment trap sampling in the estimation of POC flux. For example, there are now multiple approaches for quantifying POC from imagery across a range of particle types and sizes (e.g., Iversen et al. 2010; Briggs et al. 2011; Cetinić et al. 2012; Smith et al. 2013; Dall’Olmo and Mork 2014).

The need for spatially distributed carbon cycling observations is as great as ever. The influence of global warming is not uniform such as in coastal upwelling regions where increasing wind stress and mixing is causing increased primary production resulting in higher fluxes of particulate matter reaching abyssal depths. In more oligotrophic central gyre areas, increasing surface temperatures are predicted to increase stratification and reduce the exchange of nutrients from deeper water to fuel primary production. Reduced nutrient exchange will increase the importance of picoplankton which will limit the primary production exiting the euphotic zone and reaching the deep ocean (Doney et al. 2012; Chavez et al. 2011; Polovina et al. 2008; Jones et al. 2013).

In situ measurements of SCOC are considered reliable for estimating carbon demand by the sediment community in many regions of the ocean. However, such sensing systems to make high-quality and high-resolution measurements are more complex and costly than sediment traps. That is perhaps why there have been relatively few such measurements made over the past several decades. It should be recognized, however, that the real cost difference between sediment trap and Rover data is not very large considering that the ship-time to service either sediment traps or Rover systems far outweighs the cost of the equipment, and relevant technical support over the instrument lifetime.

## Conclusions

High-resolution measurements of SCOC are critical to developing a realistic carbon cycle model for the open ocean. The results here provide a unique perspective into variations of POC flux and SCOC that can provide new model parameters for long-term climate projections. Such input is essential to evaluate the impact of climate change on the oceanic carbon cycle, and the long-term influences on the sedimentation record. The influence of global warming is not uniform. In coastal upwelling regions, wind stress and mixing are increasing, causing increased primary production resulting in higher fluxes of particulate matter reaching abyssal depths. The highest peaks in POC flux and SCOC at Sta. M have occurred since 2011 with more calendar-months approaching or exceeding POC flux:SCOC unity on average (Fig. 4). In more oligotrophic central gyre areas, increasing surface temperatures are predicted to increase stratification and reduce the exchange of nutrients from deeper water to fuel primary production (Polovina et al. 2008; Doney et al. 2012). Reduced nutrient exchange will increase the importance of picoplankton which will limit the primary production exiting the euphotic zone and reaching the deep ocean.

Investments in instrumentation to perform these measurements over greater geographic scales and depth ranges are required for global assessments of abyssal seafloor carbon consumption and burial. The development and proven reliability of Rover II as a platform for long-term measurements of SCOC using chambers, in addition to potential microprofilers and eddy covariance (e.g., Donis et al. 2016) now allow such instruments to be deployed in other time-series studies worldwide.

## Electronic supplementary material

Below is the link to the electronic supplementary material.

Supplementary Table 1 Monthly averages of sediment community oxygen consumption (SCOC) recorded at Station M, from all instruments (XLS 70 kb)
